# Is PEEK cage better than titanium cage in anterior cervical discectomy and fusion surgery? A meta-analysis

**DOI:** 10.1186/s12891-016-1234-1

**Published:** 2016-09-01

**Authors:** Zhi-jun Li, Yao Wang, Gui-jun Xu, Peng Tian

**Affiliations:** 1 Department of Orthopedics, Tianjin Medical University General Hospital, No.154, Anshan Road, Tianjin, 300052 People’s Republic of China; 2 Department of oncological surgery, Tianjin Nankai Hospital, Tianjin Integrated Traditional Chinese and Western Medicine Hospital, No.6 Changjiang Road, Tianjin, 300100 People’s Republic of China; 3 Department of Orthopedics, Tianjin Hospital, No. 406, Jiefang Nan Road, Tianjin, 300211 People’s Republic of China

**Keywords:** Titanium, Polyetheretherketone, Cage, Cervical spine, Meta-analysis

## Abstract

**Background:**

This meta-analysis was performed to identify the benefits and disadvantages of the PEEK cage and titanium cage.

**Methods:**

We used “cervical or cervicle”, “titanium”, and “polyetheretherketone or PEEK” as keywords. Medline, Embase, Cochrane Central Register of Controlled Trials and other databases were searched to identify eligible studies that were published before October 2015. In addition, the Google search engine was used to manually search for relevant journals or conference proceedings. Randomized controlled trials and non-randomized controlled trials that compared the PEEK cage and titanium cage for anterior cervical surgery were included. The meta-analysis was performed with RevMan 5.1 software.

**Results:**

Two randomized and two non-randomized clinical trials were retrieved with a total of 184 segments from 107 patients in the PEEK cage group and 211 segments from 128 patients in the titanium cage group. The quality assessment scores ranged from 16 to 18 with high heterogeneity. There were no differences in functional status according to the Odom criteria, fusion rate, final local segmental angle and loss of correction between the two groups. Although more subsidence occurred in the titanium cage group, the effects of loss of the local segmental angle or the whole cervical Cobb angle on cervical function in the long-term are still not clear.

**Conclusion:**

The present meta-analysis indicated no significant difference in functional and radiographic performance between the PEEK and titanium cages, although more subsidence occurred in the titanium cage group. More high-quality studies are needed to confirm these results to offer more information for the choice in clinical practice.

**Electronic supplementary material:**

The online version of this article (doi:10.1186/s12891-016-1234-1) contains supplementary material, which is available to authorized users.

## Background

Anterior cervical discectomy and fusion (ACDF), introduced by Cloward [1], has been accepted as the standard procedure for the treatment of myelopathy and radiculopathy in the cervical spine [2, 3]. A tricortical iliac crest bone graft is the traditional inter-body fusion material that can show perfect bony fusion and maintain the patency of the neuroforamen. However, donor site complications were reported in fusion with an iliac bone graft, such as subcutaneous hematomas, infections, and chronic wound pain [4].

To immobilize the unstable motion segment after discectomy, we have to ensure bony fusion and avoid donor site complications at the same time; some fusion devices have been developed for stand-alone use or use in combination with an anterior plate. As described by Bagby [5], cage fusion technology originated from a surgery by Bagby, some veterinary surgeons and the distraction compression method and was the basic principle for stand-alone intervertebral cage fusion. Although the principle was invented to solve a cervical problem, the carbon fiber fusion cage [6] and titanium cage [7] were first used for lumbar inter-body fusions, and they were then applied to treat cervical spinal degenerative lesions by *Hacker* [8] and *Profeta* [9] in 2000. Currently, the titanium cage and polyetheretherketone (PEEK) cage are the two most common cages in clinical practice. The ideal cage has to have a high fusion rate and prevent complications, such as subsidence and loss of correction.

Even though a titanium cage can provide long-term stabilization, increase lordosis, and increase foramina height compared with the iliac bone graft [10], some inferior clinical outcomes appeared in clinical practice. Loss of correction is a major complication of subsidence that may eventually affect cervical spinal function after the operation. The incidence of subsidence for the titanium cage varied as reported by Gercek who retrospectively reviewed eight patients who received ACDF with a stand-alone titanium cervical cage and found that five of the nine fused levels had radiological signs of cage subsidence [11]. The subsidence was influenced by many factors, of which an important one is the higher elasticity modulus of the titanium cage. A modulus of elasticity close to cortical bone might contribute to advantages in stress distribution and load sharing, which can contribute to a lower subsidence rate and, thus, better clinical results, making PEEK cages more welcomed by surgeons.

Studies comparing titanium and PEEK cages for the treatment of cervical disc degenerative disease are rarely in the literature. Chou’s team retrospectively compared the results of anterior cervical fusion using titanium cages, PEEK cages and tricortical bone grafts [12]. They noticed a better fusion rate and less subsidence in the PEEK cages group. However, the study only enrolled a small number of patients and cervical spinal function was not evaluated. In a systematic review by Kersten who compared a PEEK cage with a bone graft, titanium cage, and carbon fiber cage, no difference was found between PEEK and titanium cage [13]. Therefore, our present study was conducted to critically review and summarize the literature to compare the results of a PEEK cage with a titanium cage for the treatment of cervical degenerative disorders to identify the better choice for the surgeons.

## Methods

### Search strategy

Electronic searches of the Medline, Embase, Cochrane Central Register of Controlled Trials and other internet databases were performed to identify trials according to the Cochrane Collaboration guidelines. The searches included literature dating from the database origin to October 2015. We used the following search terms: “cervical”, “titanium”, “polyetheretherketone” or “PEEK”. In addition, the Google search engine was searched manually using the same search terms to seek further relevant studies that may have been missed. Manual searches, including those of reference lists from all of the included studies, were used to identify trials that the electronic search may have failed to identify. There was no restriction on language. Two reviewers (Xu and Li) independently assessed the titles and abstracts of all of the reports identified by the electronic and manual searches. When inclusion was unclear based on the abstracts, the full text articles were retrieved to select those that met the eligibility criteria. We corresponded with the main authors via email to gain more data for the studies without enough details. A final confirmation of the identified studies was conducted before the meta-analysis. Any disagreements were resolved through discussion.

### Selection criteria and quality assessment

Investigations that met the eligibility criteria were included in final meta-analysis; these criteria were the following characteristics: (1) the investigation compared a PEEK cage with a titanium cage for anterior cervical surgery, (2) it was a randomized or non-randomized clinical trial, and (3) the full text article provided enough data for extraction and further analysis. We excluded articles that were duplicate reports of earlier trials, post-hoc analyses of randomized controlled trial (RCT) data and articles for which we were unable to obtain the full text. Articles involving anterior plate fixation or factors to promote osteogenesis, such as BMP, were excluded. To assess the methodological quality of the randomized trials, the review authors (Xu and Li) used a modified version of the generic evaluation tool used by the Cochrane Bone, Joint and Muscle Trauma Group [14]. The methodological index for non-randomized studies (MINORS) form was used for non-randomized clinical trials [15]. The methodological quality of each trial was scored and ranged from 0 to 24.

### Data extraction

Two authors (Xu and Li) independently extracted data from the included articles. Information regarding the study design, patient demographics, inclusion and exclusion criteria, interventions, outcomes, follow-up duration, and the rate of loss for each treatment group were extracted. We attempted to contact the main authors for Additional file 1 when the reported data were inadequate. Disagreements were resolved by consensus or consultation with the senior reviewer.

### Data synthesis and analysis

The meta-analysis was undertaken using RevMan 5.1 for Windows (Cochrane Collaboration, Oxford, United Kingdom). Statistical heterogeneity was assessed using a standard chi-square test (statistical heterogeneity was considered significant at *p* < 0.05) and the *I*^2^ statistic (*I*^2^ value of 50 % or higher was considered to indicate substantial heterogeneity) [16]. I^2^ > 50 % and *P* < 0.1 were considered to indicate significant heterogeneity. A random-effects model was applied for data analysis when significant heterogeneity was found. A fixed-effects model was used when no significant heterogeneity was found. [17]. Odds ratios (OR) and 95 % confidence intervals (CI) were calculated for dichotomous outcomes, while mean difference (MD) and 95 % CI were calculated for continuous outcomes.

## Results

### Study characteristics

**Fig. 1:**
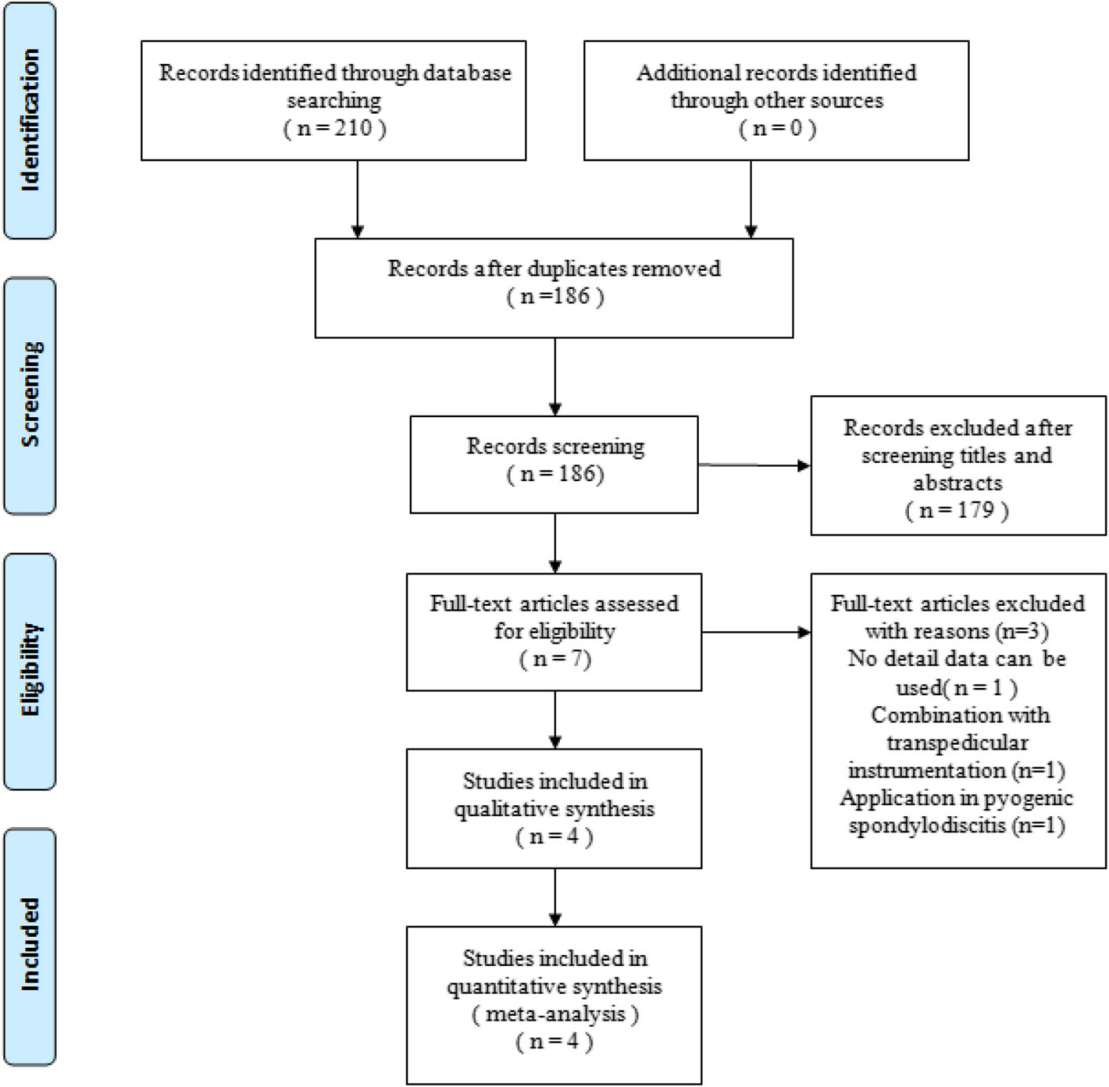
PRISMA flowchart of studies

Figure 1 shows the search strategy of the study selection and inclusion process for the study. Among the search results, 179 studies did not compare titanium and PEEK cages, one study did not provide detailed data, one study used transpedicular instrumentation, and another study used cages for pyogenic spondylodiscitis. There were 2 randomized clinical trials [18, 19] and 2 non-randomized clinical trials [12, 20] that satisfied the pre-defined inclusion criteria and were included in this study. Individual patient data were available from these articles without the data for those lost to follow-up.

Characteristics of the included studies are summarized in Table 1. These studies involved 184 segments from 107 patients in the PEEK cage group and 211 segments from 128 patients in the titanium cage group. Most baseline parameters were comparable except that the patients of the PEEK cage group were older than the titanium cage group in the study by Cabraja et al. [20]. The patients in the two groups were not contemporary, with a 3 year difference in the study by Chou et al. [12]. The study by Chen et al. [18] aimed to compare outcomes of titanium and PEEK cages for the treatment of three levels of cervical spondylotic myelopathy with a 7-year follow-up. The sample size was not calculated prospectively in all studies. There were 11 patients in the titanium group and 9 in the PEEK cage group that were lost at the final follow-up in the study by Chen et al. However, the two groups were comparable in baseline parameters such as age, gender, operated segments and follow-up time.

**Table 1:** Characteristics of included studies

study	group	Gender (M/F)	Age(Y)	Segments (one/two/three)	Diseases(T/P)	Cage information	Follow-up(m)
Chou YC et al. 2008 [12]	TTN	11/16	55.2	43(14/10/3)	Trauma(1/2) spondylosis(10/0) OPLL(1/0) HIVD(15/7)	Non-threaded cage containing a biphasic calcium phosphate ceramic	12
	PEEK	6/3	54.2	15(3/6/0)		containing a biphasic calcium phosphate ceramic	12
Niu CC et al. 2010 [19]	TTN	15/13	49.5 ± 11.3	37(19/9/0)	Radiculopathy(21/9) Myelopathy(3/3) Radiculomyelopathy(4/3)	hydroxyapatite-coated, box-shaped device with a tooth-threaded surface filled with a local bone graft and a calcium phosphate bone substitute	31.9 ± 3.4
	PEEK	12/13	52.2 ± 10.5	34(16/9/0)		filled with allo-cancellous bone graft	30.4 ± 3.3
Cabraja M et al. 2012 [20]	TTN	26/18	51.1 ± 8.9	44(1/0/0)	Radiculopathy(36/34) Myelopathy(8/8)	with Plasmapore coating	30.6 ± 14.3
	PEEK	28/14	57.6 ± 11.1	42(1/0/0)		N	26.1 ± 10.0
Chen Y et al. 2013 [22]	TTN	17/12	45.7 ± 7.2	87(0/0/3)	Radiculopathy(16/18) Myelopathy(1/1) Radiculomyelopathy(12/12)	N	97.2
	PEEK	16/15	47.2 ± 6.8	93(0/0/3)		N	102.1

Notes: *TTN*, Titanium, *PEEK* polyetheretherketone, *OPLL* ossification of the posterior longitudinal ligament, *HIVD* herniated intervertebral disc, *N* non-mentioned

### Risk of bias assessment

There are some weaknesses in the methodological design of the included studies that we must keep in mind. Details about the methodological quality of the included studies are listed in Table 2. Their quality score ranged from 16 to 18. The unclear blindness was a major problem for the included studies. The outcome of assessors’ blindness was only described in the study by Niu et al. The participants’ and treatment providers’ blindness was not clear in the studies by Niu et al. and Chen et al. There was no prospective calculation of the sample size and no data collection in the two non-randomized trials.

**Table 2:** Details about methodological quality of included studies

Quality assessment for randomized trials	Niu CC	Chen Y	Chou YC	Cabraja M	Quality assessment for non-randomized trials
Was the assigned treatment adequately concealed prior to allocation?	2	1	2	2	A clearly stated aim
Were the outcomes of participants who withdrew described and included in the analysis?	0	2	1	1	Inclusion of consecutive patients
Were the outcome assessors blinded to treatment status?	2	0	0	0	Prospective data collection
Were the treatment and control group comparable at entry?	2	2	1	2	Endpoints appropriate to the aim of the study
Were the participants blind to assignment status after allocation?	0	0	1	2	Unbiased assessment of the study endpoint
Were the treatment providers blind to assignment status?	0	0	2	2	A follow-up period appropriate to the aims of the study
Were care programmes, other than the trial options, identical?	2	2	2	2	Less than 5 % loss to follow-up
Were the inclusion and exclusion criteria clearly defined?	2	2	0	0	Prospective calculation of the sample size
Were the interventions clearly defined?	2	2	2	2	Prospective calculation of the sample size
Were the outcome measures used clearly defined?	2	2	1	2	An adequate control group
Were diagnostic tests used in outcome assessment clinically useful?	2	2	2	1	Baseline equivalence of groups
Was the surveillance active, and of clinically appropriate duration?	2	2	2	2	Adequate statistical analyses

### Meta-analysis results

#### Clinical functional status

**Fig. 2:**
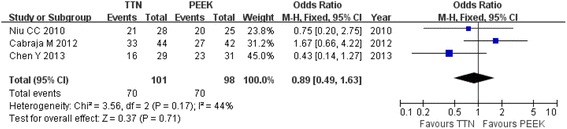
Forest plot showing clinical functional status by Odom criteria

Three studies evaluated clinical function using the Odom criteria [18–20]. There were no significant differences between the two groups (OR = 0.89; 95 % CI: 0.49 to 1.63; *P* =0.71; *I*^2^ = 44 %; Fig. 2). There was similar postoperative cervical function in the studies by Niu et al. [19] (75 % vs. 80 %, respectively; *P* = 0.664) and Cabraja et al. [20] (75 % vs. 64.3 %, respectively; *P* = 0.395). Chen et al. reported better results from the PEEK cage group than the results from the titanium cage group (16/29 vs. 23/31, respectively). Clinical performance measured by the neck disability index (NDI) was reported by Cabraja et al. and Chen et al. [18], but a comparison was not performed because of the significant difference in follow-up duration. Cabraja et al. showed that the final NDI was 16.89 ± 10.24 in the titanium cage group, while it was 17.05 ± 9.6 in the PEEK cage group without a significant difference (*P* = 0.940). The final NDI was 21.6 ± 2.6 and 15.2 ± 2.3 in the titanium and PEEK cage groups, respectively; this was a significant difference (*P* < 0.05) in the study by Chen et al. The final JOA score was also reported by Chen et al. and indicated that the PEEK cage group was better (14.2 ± 1.8) than the titanium cage group (12.8 ± 1.8).

### Radiological performance

**Fig. 3:**
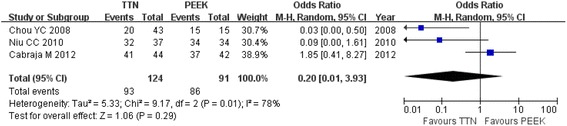
Forest plot showing fusion rate between two groups

The fusion rate was reported in all of the studies. Chen et al. reported that all patients achieved fusion at the final 7-year follow-up, but it was not included in this meta-analysis because of the long-term follow-up. In the other three studies, 31 of the 124 segments in the titanium cage group did not show fusion at 12 months, while 5 of the 91 segments in the PEEK cage group did not show fusion at 12 months. The pooled data were analyzed using a random-effect model because heterogeneity existed (*I*^2^ = 78 %). There was no significant difference between the two groups (OR = 0.2; 95 % CI: 0.01 to 3.93; *P* = 0.29; Fig. 3). For the titanium cage group, a significantly lower fusion rate of 46.51 % was noticed at 12 months in the study by Chou et al. [12]. The fusion rate was lower in the PEEK cage group compared with the titanium cage group (88.1 % vs. 93.2 %, respectively) in the study by Cabraja but the comparison was not significantly different.

**Fig. 4:**
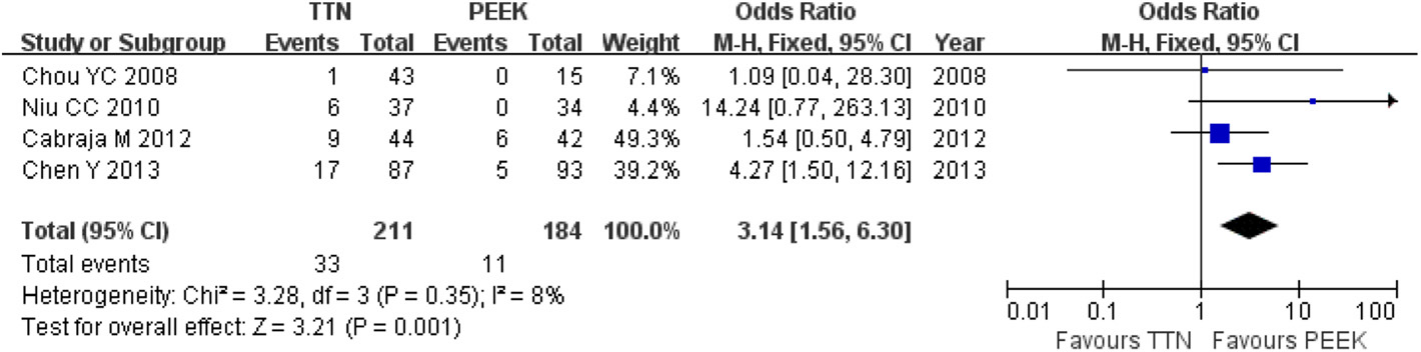
Forest plot showing subsidence between two groups

Subsidence was reported in all of the studies among 33 of the 211 patients in the titanium cage group and among 11 of the 184 in the PEEK cage group. There was a significant difference between the two groups (OR = 3.14; 95 % CI: 1.56 to 6.30; *P* = 0.001; Fig. 4). When we excluded the data from Chen’s study because of their long-term follow-up, there were many more cases of subsidence in the titanium cage group (OR = 2.4; 95 % CI: 0.93 to 6.18; *P* =0.07).

**Fig. 5:**
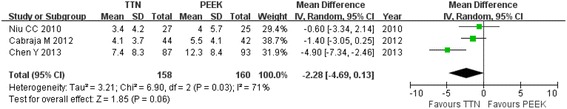
Forest plot showing the final segmental angle between two groups

The final cervical angle and loss of correction were reported in three studies [18–20]. The methods used to measure the cervical angle were different in these articles. For the segmental angle at the surgery level, there was no significant difference between the two groups with high heterogeneity (MD = −2.28; 95 % CI: −4.69 to 0.13; *P* =0.06; *I*^2^ = 71 %; Fig. 5). Excluding the results from Chen et al., heterogeneity decreased to 0 but did not show significant difference (MD = −1.19; 95 % CI: −2.60 to 0.23; *P* =0.10). The loss of segmental correction was similar between the two groups (MD = 2.03; 95 % CI: −1.10 to 5.17; *P* =0.20; *I*^2^ = 94 %). The cervical angle was measured by using the Cobb angle from C2-C7 in two studies. In the study by Cabraja, there was no significant difference between the titanium and PEEK cage groups (13.1 ± 12.7 vs. 13.3 ± 7.1, respectively; *P* = 0.288). No data about the loss of Cobb angle from C2-C7 was reported in that article. In contrast, the PEEK cage group showed better a Cobb angle from C2-C7 than the titanium cage group (13.65 ± 8.92 vs. 7.86 ± 8.52, respectively; *P* <0.05) in the study by Chen et al. The loss of this angle was greater in the titanium cage group than that of the PEEK cage group (8.59 ± 4.67 vs. 4.84 ± 2.39, respectively; *P* <0.05).

### Other complications

In addition to subsidence and loss of correction, other complications were also reported. There was 1 instance of limb numbness, 1 instance of neuropathic problem, 1 instance of weakness, and 1 instance of subluxation appeared in the titanium cage group, while there was 1 instance of wound pain in the PEEK cage group in the study by Chou. There were also 2 cases of dislocation reported in the titanium cage group by Chen.

## Discussion

Titanium and PEEK cages are commonly used cages for the anterior cervical discectomy and fusion. Our present meta-analysis was conducted to compare the outcomes of both types of cages after ACDF. The results showed a similar fusion rate, loss of correction at the surgery segment, and clinical function by the Odom criteria between the titanium and PEEK cages. Although the incidence of subsidence was higher in the titanium cage group as calculated from the data for all of the studies, it changed after excluding the data from Chen’s study. It seems that the titanium and PEEK cages perform similarly when used for ACDF. However, there are more considerations that should be taken when we are interpreting these results.

No matter whether a type of cage or iliac crest bone is adopted, the important aim was to realize that solid fusion is the basic foundation to maintain good clinical function. A tricortical iliac crest bone graft had been considered as the golden standard for good fusion [21]. A titanium cage can also maintain fusion well because it was first used for this practice. According to the literature, the fusion rate of the titanium cage was 84 % by Yang et al. [22], 95 by Moreland et al. [23], and even 98 % by Schmieder et al. [24]. It is hard to believe that the fusion rate was only 46.51 % at 12 months in the included study by Chou et al. There was no clear explanation for this in Chou’s study. PEEK is a biocompatible material with many perfect qualities for this application, such as a corrosion resistant ability [25], the absence of cytotoxicity and mutagenicity [26] and a close elasticity modulus to bone. Based on these material characteristics, the PEEK cage have been used in ACDF with better fusion rate as reported 94 by Hwang et al. [27] and 100 % by both Cho et al. [28] and Niu et al. [19]. Most of these studies had a relatively short follow-up. In the longest 7-year observation by Chen et al., all of the patients in both groups achieved bony fusion [18]. Therefore, we hypothesized that the cage material only has an effect on the fusion rate for a short time after ACDF.

Based on the Odom criteria, similar postoperative cervical function was observed in the included studies by Niu and Cabraja. However, Chen reported better results in the PEEK cage group than that of the titanium cage group after their long follow-up observation. The patients enrolled into these three studies were different. The patients received a single level ACDF in the study by Cabraja. Most of patients in the study by *Niu* received a single level ACDF and some of patients received a two level ACDF. It was significantly different that a three level ACDF was offered to all of the patients in the study by *Chen* et al. Limited by the few studies that compared titanium and PEEK cages, however, we cannot draw a conclusion that PEEK cages performed better in multiple level ACDF over a long time.

Subsidence was a common follow-up observation that can lead to deterioration of the long-term function. The incidence of cage subsidence for the titanium cages ranged from 13 to 62.5 % for cases in the literature [11, 12, 18–20, 24, 29]. Benefitted by the better material property, the PEEK cage shows less subsidence, varying from 0 to 18 % [18–20, 30–32], which is consistent with our result that less cage subsidence was observed in the PEEK cage group. However, some authors assumed that the cage subsidence was not affected by the differences in the modulus of material elasticity [12, 19]. In addition to the cage material, many risk factors, including suboptimal surgical techniques and parameters of the cage [33], may result in increased stress of the endplates and, thus, increased risk of subsidence if the bone mineral density of the vertebral body is not strong enough to bear it [34, 35]. The patients of the PEEK cage group were older than those of the titanium cage group in the included study by Cabraja [20]. Patients in the PEEK cage group may suffer more from osteoporosis with a higher risk of cage subsidence.

Subsidence will a cause loss of correction of the segmental angle and the Cobb angle from C2-C7. Loss of cervical lordosis is a risk factor that contributes to degeneration in the adjacent segments [36, 37]. Kyphotic malalignment changes the dynamic kinematics of the cervical spine and accelerates the degenerative process [38]. It is the opinion of others that subsidence less than 2 mm into the vertebral bodies until fusion is acceptable [39]. Barsa et al. found that subsidence occurred during early follow-up and no evidence of progression appeared beyond 3 months [29]. There were no radiographic signs of progressive degenerative changes in any of the adjacent segments during the 2-year follow-up. Wu and his colleagues reported that cage subsidence did not exert a significant impact upon the long-term clinical outcomes [40]. The study by Chen et al. did not provide evidence of radiological and clinical progression after the long-term follow-up [18]. Therefore, the actual effect of loss of correction on long-term function is still unclear.

Some limitations must be clarified about our present work. The results provided in this article came from two RCTs and two non-RCTs, and they were analyzed together. The power of our meta-analysis is limited by the small number of high-quality RCTs. The four included studies were different in cages, levels of ACDF, and follow-up time. Although we tried our best to summarize and analyze the data, high heterogeneity existed without a subgroup analysis. Therefore, more high-quality RCTs are needed to improve the methodology, to minimize bias and to confirm the effect of these two types of cages on long-term radiographic performance and clinical function.

## Conclusion

The results of this review show that titanium and PEEK cages perform similarly in attaining bony fusion and maintaining the clinical function in the anterior cervical decompression and fusion. Although more subsidence occurred in the titanium cage group, the effects of loss of local segmental angle or the whole cervical Cobb angle on cervical function in the long-term are still not clear. More high-quality, randomized controlled trials are required to further understand the application of cages in ACDF.

## Additional file

Additional file 1: Raw data of RevMan 5.1 for Windows. (XLS 27 kb)

## Abbreviations

ACDF: Anterior cervical discectomy and fusion
CI: Confidence interval
MINORS: Methodological index for non-randomized studies
MD: Mean difference
NDI: Neck disability index
OR: Odds ratios
PEEK: Polyetheretherketone
RCTs: Randomized controlled trials.
